# Investigation and source-tracing of an anthrax outbreak in Gansu Province, China

**DOI:** 10.1371/journal.pone.0203267

**Published:** 2018-08-30

**Authors:** Deshan Yu, Jian He, Enmin Zhang, Peng Wang, Dongpeng Liu, Yadong Hou, Huimin Zhang, Kongfu Wei, Faxiang Gou, Huijuan Zhang, Wei Li, Jianchun Wei

**Affiliations:** 1 Gansu Provincial Center for Disease Control and Prevention, Lanzhou, Gansu Province, China; 2 National Institute for Communicable Disease Control and Prevention, China CDC, Beijing, China; 3 State Key Laboratory for Infectious Disease Prevention and Control, Beijing, China; 4 Collaborative Innovation Center for Diagnosis and Treatment of Infectious Disease, Hangzhou, China; 5 Min County Center for Disease Control and Prevention, Minxian, Gansu Province, China; CCAC, UNITED STATES

## Abstract

Anthrax is an endemic disease in China. Cases are reported every year, especially in the northwestern areas. In August 2016, an outbreak of 21 cutaneous anthrax cases was reported in Min County, Gansu Province, China. In this study, the general characteristics of the anthrax outbreak are described. Two molecular typing methods, canonical single-nucleotide polymorphism (canSNP) and multiple-locus variable-number tandem repeat analysis with 15 markers (MLVA15), were used to investigate the possible source of transmission and to identify the genetic relationship among the strains/samples isolated in this outbreak as well as previous isolates. In this outbreak, all patients were infected through contact with diseased livestock or contaminated animal products. Livestock had been introduced into the local area shortly before the outbreak from Gannan Prefecture (in Gansu Province), Sichuan and Qinghai Provinces. In the molecular typing analysis, there were two canSNP subgroups found in Gansu, A.Br.001/002 and A.Br.Ames, and five MLVA15 genotypes were observed. The strains collected from the anthrax outbreak in Min County in 2016 belonged to the A.Br.001/002 canSNP subgroup and the MLVA15-28 and MLVA15-30 genotype. Strains previously isolated from Sichuan, Inner Mongolia and Maqu County (in Gannan Prefecture, Gansu Province) were clustered with these outbreak-related strains/samples according to the MLVA15-30 genotype. The MLVA15-28 genotype was found in strains isolated from Gansu and Xinjiang in previous studies. Combining the epidemiological investigation and molecular typing results, we conclude that the patients in this outbreak were infected by a local pathogen present in the adjoining area of Gansu, Sichuan and Qinghai Provinces.

## Introduction

Anthrax is primarily a disease of herbivores; animals become infected through the uptake of pathogenic spores from the environment. Human are usually infected by contact with infected animals or contaminated animal products. Depending on the route of infection, there are three primary forms of anthrax in humans: inhalational, gastrointestinal and cutaneous [1]. Approximately 95% of infections are cutaneous, which is mainly caused by the handling of infected animal carcasses or the products of diseased animals [2,3].

*Bacillus anthracis* (*B*. *anthracis*), the causative agent of anthrax, is a relatively homogeneous bacterial species; its lack of polymorphisms may be due to its life cycle, which includes long periods of time as dormant endospores [4]. Genetic markers, such as single-nucleotide polymorphisms (SNPs) and variable-number tandem repeats (MLVA), have been used to characterize the phylogenetic and evolutionary relationships of *B*. *anthracis* strains [5–7] and have been used as source-tracing methods in the event of anthrax outbreaks or bioterrorist attacks [8–10]. The global genetic population structure of *B*. *anthracis* has previously been defined by canSNP and MLVA analysis, in which, the majority strains of A.Br.001/002 sub-group (70%) in the world and most of the diversity for this sub-group were sourced from Chinese *B*.*anthracis* [4].

Anthrax is a global disease. The incidence of anthrax has been reduced by continuous research and disease control measures, but it still occurs in undeveloped and developing countries [10]. Anthrax cases are reported every year in China, especially in the northwestern provinces in mainland China [11]. Gansu Province, located in the northwest of China, is adjacent to Inner Mongolia, Xinjiang, Sichuan, Qinghai and Shaanxi Provinces. Over the past two decades, there have been cases reported in Gansu Province every year. From 2007–2016, the annual number of anthrax cases ranged from 19 to 82 and accounted for 6.57% to 24.31% of the total number of cases in China (Table 1).

**Table 1:** Cases of human anthrax in Gansu Province, 2007–2016.

Year	Population (million)	No.of cases	Percent in China (%)
**2007**	25.48	43	10.21
**2008**	25.51	45	13.39
**2009**	25.55	46	13.11
**2010**	25.60	19	6.57
**2011**	25.64	39	12.62
**2012**	25.78	23	9.70
**2013**	25.82	41	21.24
**2014**	25.91	55	22.18
**2015**	26.00	70	24.31
**2016**	26.10	82	21.93

In August 2016, an anthrax outbreak, including twenty-one cutaneous cases, was reported in Gansu Province. To determine the relationship among the cases and investigate the infectious sources and possible routes of transmission, epidemiologic investigations and laboratory investigations were performed by the local CDC and China CDC.

## Methods

### Ethics statement

This study was reviewed and approved by the Ethical Committee [Institutional Review Board (IRB)] of National Institute for Communicable Disease Control and Prevention, China CDC (License number: ICDC-2014013). All adult subjects provided informed consent, and a parent or guardian of any child participant provided informed consent on their behalf. The informed consent was given orally for all participants, as this is usual practice in anthrax outbreak investigations, and oral consent is also a safe manner to minimize the risk of contamination since most of patients have lesions on their arms or hands. The consents were recorded in daily progress notes by the attending physician in the local hospital of Gansu Province. The IRB approved the use of oral consent, and the consent information contained the aim of the study, the usage of the patient’s samples and other information. No live animals were euthanized in the study, and the samples were collected from dead animals with permission from the animal owners.

### Epidemiological investigation

In the study, the anthrax outbreak is defined as 10 or more cases with common exposure history or in a residential community (a village, a school, a factory, etc.) occurring within two weeks. In August 2016, a suspected anthrax outbreak was reported to China's National Health and Family Planning Commission. A field team, including clinical, epidemiologic and laboratory personnel, traveled to the outbreak site to perform investigations and undertake disease control measures. The epidemiologist searched for suspected cases of anthrax in affected areas and investigated their exposure history. The diagnosis of case was based on characteristic clinical manifestations in combination with history of exposure and supplemented by laboratory tests. A suspected case of cutaneous anthrax in humans was defined as a patient who had acute onset of a skin lesion with vesicles or ulceration with a raised margin and central black eschar with epidemiological exposure. Cases were confirmed through supportive laboratory tests, including isolation of *B*. *anthracis* and a ≥ 4-fold increase in specific antibody titers against *B*. *anthracis*. All human anthrax cases were diagnosed according to the unified case definitions issued by the Chinese Ministry of Health in 2008.

### Laboratory diagnosis

Specimens from patients, animals and the environment were collected for laboratory diagnosis. Blister fluid and sera were collected from all patients. Sera were collected from several patients three times. The first serum specimens were collected when patients first sought medical advice. Nineteen of twenty-one initial serum specimens were collected in the 10 days after the onset of symptoms, and 2 were collected approximately 20 days after the onset of symptoms. Most of the second serum specimens were collected between 2 and 6 weeks after the onset of symptoms. The third serum specimens were collected after 5 months.

Culturing, DNA detection by PCR and antibody detection by ELISA were applied in the outbreak. Blister fluid from patients and samples from animals and environment were streaked onto nutrient LB agar plates or blood agar plates for culture. The bacteria isolated from samples were identified by culture, microscopic examination, bacteriophage lysis test and real-time PCR. DNA extraction and PCR amplification were performed as described previously [12]. The bacterial culture and DNA preparation were performed in a Biosafety level 3 (BSL-3) Laboratory. Sera were analyzed by ELISA to identify anti-protective antigen (PA) antibody titers. ELISA procedures were performed as described by Quinn et al. [13], with the minor modification that the result was recorded as an antibody titer. A four-fold rise in anti-PA antibody or positive conversion was used as criteria to confirm infection.

### Molecular genotyping

Two strains from patients, two strains from dead sheep and 6 blister fluid samples from suspected anthrax patients in the outbreak were analyzed. In addition, 6 *B*. *anthracis* strains collected from 1954–2016 in Gansu Province, and 37 *B*. *anthracis* strains collected from other provinces (8 from Xinjiang, 8 from Sichuan, 1 from Qinghai, 17 from Inner Mongolia, 2 from Shaanxi and the A16R vaccine strain) were also included in the study.

The canSNP analysis and MLVA15 analysis were performed as described previously [4,12]. The nomenclature of canSNP subgroups and MLVA15 genotypes described by Van Ert et al. [4] was used. Data from MLVA analysis were imported into the BioNumerics software (version 5.10, Applied-Maths) and were processed by clustering analysis using the categorical coefficient and the unweighted pair-group method with arithmetic means. Cluster analysis of the categorical data was presented using dendrograms.

## Results

### Anthrax outbreak description

**Fig 1:**
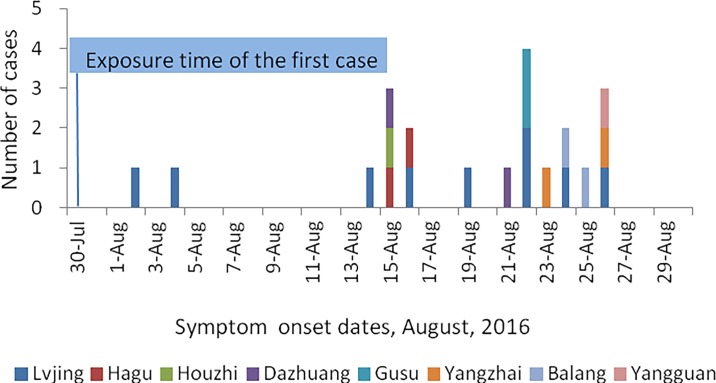
Onset time of patients in Min County, Gansu Province, 2016. The villages where the patients were found are indicated by different colors.

In August 2016, an outbreak of 21 suspected cutaneous anthrax cases was reported by the health authorities of Min County, Gansu Province. These patients were scattered in eight villages (nine in Lvjing, two in Dazhuang, two in Hagu, two in Balang, two in Yangzhai, two in Gusu, one in Houzhi and one in Yangguan) and were associated with at least three exposure events (Fig 1). None of the patients died. The index case (male, 66 years old), who butchered a sick sheep on July 30 with his wife, suffered cutaneous anthrax on fingers on August 2, and his wife onsets on August 4. The onset dates of the 21 cases ranged from August 2 to August 26, and the mean duration between exposure and onset of symptoms was 3.5 days (range: 1–10 days). The median age of the patients was 37 years (range: 12–64 years). Nineteen (90%) patients were men, and two were women. Fifteen cases were farmers, four cases were herdsman, one was a student, and one was a catering worker. All 21 cases had exposure history to cattle, sheep or animal products.

The disease in animals was also investigated. Min County is a traditional trading place for livestock and their products in Gansu Province, where livestock are rarely vaccinated, and many people engage in livestock farming, trade and butchering. Several cattle and sheep were introduced into Min County from Gannan Prefecture, Sichuan Province and Qinghai Province in July of 2016. From August 2 to September 12, investigators found 38 dead livestock in the outbreak area and carried out disposal according to biosafety procedures. The anthrax vaccine was mobilized and used to inoculate livestock in the affected villages after the outbreak. A total of 323,283 livestock were inoculated.

### Case diagnosis

In this study, 14 (67%) of the 21 cases met the surveillance definition for a confirmed case; all of them had elevated serum anti-PA IgG levels by ELISA, however, only 2 were confirmed by isolation of *B*. *anthracis* from a clinical specimen (from skin lesions). Anti-PA IgG could also be detected in 5 suspected patients, but these did not meet the criteria of a positive conversion or a >4-fold rise. In one patient, anti-PA IgG was not detectable at 3, 7 or 152 days after the onset of symptoms. The demographic characteristics and results of diagnostic tests are presented in Table 2.

**Table 2:** The characteristics and diagnostic testing of the 21 outbreak-related cases.

Case no.	Sex	Age	Onset date, 2016	PCR^a^	ELISA^a^			Case status
					1	2	3	
**1**	M	42	8–26	+ (5)	-(3)	NA	1:1600 (148)	Confirmed
**2**	M	51	8–14	NA	1:1600 (10)	1:400 (15)	1:800 (160)	Suspected
**3**	M	33	8–19	- (6)	1:800 (6)	1:1600 (25)	1:800 (155)	Suspected
**4**	M	30	8–23	+ (2)	- (2)	1:100 (21)	1:100 (151)	Confirmed
**5**^b^^,^^c^	M	27	8–21	+ (4)	- (4)	1:200 (23)	1:400 (153)	Confirmed
**6**	M	25	8–26	+ (3)	- (3)	1:100 (18)	1:200 (148)	Confirmed
**7**^**c**^	M	64	8–2	NA	1:3200 (23)	1:3200 (42)	1:400 (172)	Suspected
**8**	F	64	8–4	NA	1:1600 (21)	1:1600 (40)	1:800 (170)	Suspected
**9**	F	37	8–22	- (3)	- (3)	- (7)	- (152)	Suspected
**10**	M	45	8–16	- (9)	1:1600 (9)	NA	1:400 (158)	Suspected
**11**	M	64	8–25	- (4)	1:400 (4)	NA	1:1600 (149)	Confirmed
**12**	M	42	8–15	+ (7)	- (7)	1:400 (18)	1:800 (159)	Confirmed
**13**	M	40	8–15	NA	1:50 (7)	1:200 (18)	1:800 (159)	Confirmed
**14**	M	12	8–24	- (1)	1:50 (1)	1:50 (10)	1:800 (150)	Confirmed
**15**^**b**^	M	33	8–22	+ (3)	- (3)	1:100 (14)	1:200 (152)	Confirmed
**16**	M	25	8–26	+ (5)	- (3)	NA	1:1600 (148)	Confirmed
**17**	M	42	8–22	+ (7)	1:50 (7)	1:1600 (22)	NA	Confirmed
**18**	F	37	8–22	+ (7)	1:100 (7)	1:1600 (22)	NA	Confirmed
**19**	M	26	8–15	- (7)	- (7)	1:200 (18)	NA	Confirmed
**20**	M	43	8–16	+ (6)	- (6)	1:3200 (17)	NA	Confirmed
**21**	M	23	8–24	+ (5)	- (5)	NA	NA	Suspected

^a^The numbers in brackets indicate sample collection times (days after onset of symptoms).

^b^Culture is positive.

^c^*Bacillus anthracis* strains were isolated from sheep belonging to the case. NA, not available.

DNA detection by PCR was applied in the outbreak, and 11 of the 17 cases (no samples were acquired from the other 4 cases) showed evidence of *B*. *anthracis* DNA from patient tissues or lesions. In the study, there were 16 patients whose samples were identified by ELISA and PCR together. Ten of these were identified as positive and three were identified as negative by both ELISA and PCR. Three patients were found to be positive only by ELISA, and no patients who were PCR-positive and ELISA-negative were identified. Statistical analysis showed that the positive identification rate by PCR is consistent with the positive identification rate by ELISA (exact probabilities in 2x2 table, p = 0.0357).

### Genetic characteristic analysis of *B*. *anthracis* in Gansu Province

**Fig 2:**
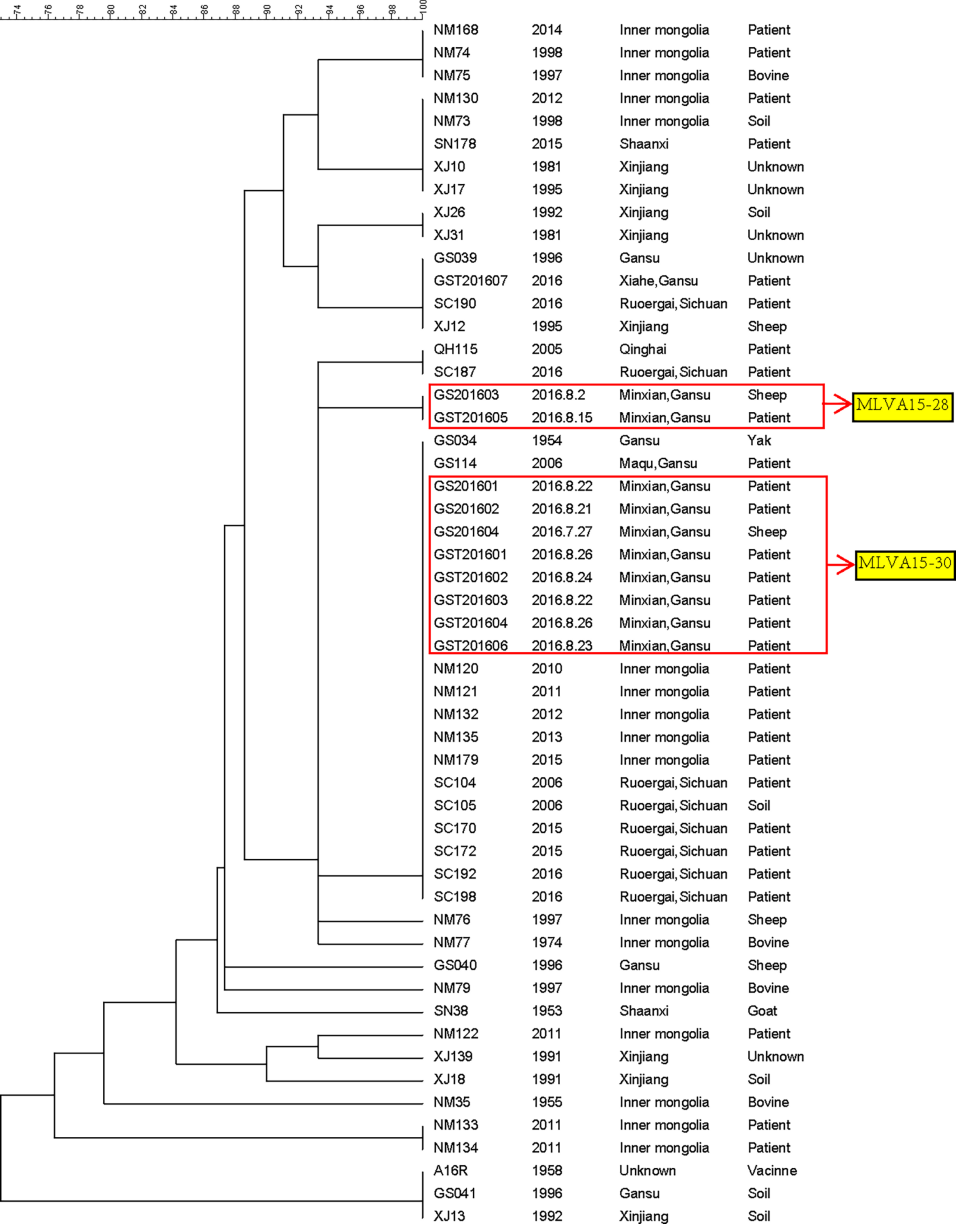
Dendrogram of MLVA15 genotypes among the isolates collected from Gansu Province and other regions.

According to the canSNP analysis, all of the strains/samples in the study were divided into 2 subgroups, A.Br.001/002 and A.Br.Ames. The outbreak-related strain/samples collected in Min County were classified as the A.Br.001/002 subgroup, which is the major canSNP subgroup in Gansu. Only one strain (from 1954) belonged to the A.Br.Ames subgroup among the strains from Gansu. MLVA15 analysis was used to subtype these strains/samples. All of the Gansu strains/samples were divided into 5 genotypes, and the outbreak-related strains/samples collected from Min County were classified as the MLVA15-30 genotype and MLVA15-28 genotype (Fig 2). The results showed that the patients in this outbreak were infected by different sources.

The samples/strains from 7 patients and 1 sheep in the outbreak were classified as MLVA15-30. Another two strains from Gansu also belonged to this genotype. In addition, the strains isolated from Sichuan (Ruoergai, 2006, 2015 and 2016) and Inner Mongolia (2010–2013, 2015) were also classified as the MLVA15-30 genotype.

Another genotype (MLVA15-28) found in the outbreak consisted of two strains, one from a patient and one from sheep. No strain with the same type was found in our samplings; however, in previous studies, strains of the same type were found in Gansu (1998, A0591CHI) and Xinjiang (A0601CHI, A0602CHI, A0603CHI, 1997) [14]. The difference between MLVA-30 and MLVA-28 is only in one VNTR locus, and the similarity is as high as 93.33%.

## Discussion

Anthrax cases are reported in Gansu Province every year. Epidemics always occur in Gannan and Linxia Prefecture, where animal husbandry dominates the local economy. Gannan Prefecture is a high-prevalence area for anthrax in Gansu, and more than 90% of anthrax cases in Gansu from 2012–2015 (Surveillance and reporting data for notifiable infectious diseases, China CDC) were from Gannan Prefecture (mainly Maqu, Luqu and Xiahe counties). No anthrax cases had been reported in Min County since 1958. From July 16 to 22, 2016, there was a Fair for Animal Production held in Lvjing, Min County. During the fair, cattle and sheep were introduced into Min County from Gannan Prefecture, Sichuan Province and Qinghai Province, mainly from Gannan Prefecture. Sichuan Province and Qinghai Province are also high-prevalence areas for anthrax. Previous studies have shown that the area at the junction of Sichuan, Gansu and Qinghai Provinces had the most livestock anthrax cases from 2005–2013 and is a potential high-risk region for the occurrence of human cases [15].

In this study, we found that the genotypes MLVA15-30 and MLVA15-28 of *B*. *anthracis* caused the outbreak. Moreover, the genotype MLVA15-30 was also responsible for the vast majority of *B*. *anthracis* infections in Gannan Prefecture and Sichuan Province. Sichuan Province is adjacent to Gansu Province, especially Ruoergai County (Aba Prefecture, Sichuan) and Maqu County (Gannan Prefecture, Gansu), which are connected by adjoining grassland. The epidemiological investigation found that the affected villages obtained cattle and sheep from Gannan and Sichuan in July 2016. According to the results of epidemiologic and laboratory analyses, the source of the outbreak was from Gannan and Sichuan, or rather the Ruoergai-Maqu grassland (Fig 1). Another genotype in the outbreak, MLVA15-28, was found in Gansu and Xinjiang in previous studies, which suggests that the epidemic related to this genotype occurred locally because no livestock were imported into the affected village shortly before the outbreak from Xinjiang. Based on our molecular typing results, we strongly suspect that the outbreak that occurred in Min County was caused by different infectious sources; however, they all originated in Gansu or Sichuan, especially the area at the junction of Sichuan, Gansu and Qinghai Provinces.

In this outbreak, high-risk exposure history, including contact with diseased livestock or contaminated animal products, was identified in all 21 cases. The skin lesions of patients and their exposure histories were the main evidence supporting clinical diagnosis. Since bacterial isolation is difficult due to the early use of antibiotics, the confirmation of anthrax infection relied on the results of anti-PA titers by ELISA. However, ELISA has certain limitations, including that it requires acute-stage serum and convalescent serum and that it takes a long time to obtain convalescent serum. In this study, there were five patients with detectable anti-PA titers, but they were unable to be defined as confirmed cases because the first serum specimens of these patients were collected after disease onset 7 days, when the anti-PA IgG was at a high level. Therefore, the sample collection time is very important for definitive results. The 7th day after symptom onset is a crucial time point. Before this time point, the positive ELISA rate is 25% (3/12); after that, the positive rate can be as high as 97.14% (34/35). According to our results, acute phase serum should be collected within 7 days of symptom onset, and convalescent serum should be collected between 2 weeks and 5 months. A previous study showed that antibodies become detectable for the first time on 12 days after symptom onset [13]; however, in our study, several serum samples collected within 7 days of symptom onset were anti-PA-positive. These cases primarily lived in remote rural areas, and most of them were farmers and herders; they usually ignore certain mild symptoms, and the actual onset date may therefore have been earlier than the reported onset date.

Throughout this investigation, there was a continuing need to develop and promote the use of sensitive testing methods. In this study, we compared ELISA and PCR methods, and our results show that the PCR method is as sensitive as ELISA and more rapid. Moreover, PCR has been listed in the anthrax diagnostic criteria of the WHO and many countries [3]; therefore, we strongly suggest that it should be added to the diagnostic criteria for anthrax in China.

Besides what have been mentioned above, there are two points need to be noticed in the outbreak. The first one is about vaccination of livestock, which was performed in the affected villages after the outbreak. Vaccination of livestock is certainly the fundamental control measure in enzootic areas. However, according to WHO, when an incident occurs unexpectedly in a non-endemic area, antibiotic treatment of exposed animals may be preferable to vaccination [3]. So vaccination of livestock is more important in the original source area, but it had been neglected in the outbreak. Another point is about public health education. The local people knew very little about anthrax; sick or dead animals were frequently slaughtered, and the meat was usually consumed or even sold by the villagers. All of the anthrax cases in the outbreak were caused either by butchering or contact with sick animals. Similar reports have frequently been found in China [12,15–19]. Therefore, strengthening public health education remains the primary measure to prevent and control anthrax in China.

## Supporting information

S1 Table The MLVA15 profiles involved in this study. (XLS)
